# House Fly (*Musca domestica* L.) Attraction to Insect Honeydew

**DOI:** 10.1371/journal.pone.0124746

**Published:** 2015-05-13

**Authors:** Kim Y. Hung, Themis J. Michailides, Jocelyn G. Millar, Astri Wayadande, Alec C. Gerry

**Affiliations:** 1 Department of Entomology, University of California Riverside, Riverside, California, United States of America; 2 Department of Entomology and Plant Pathology, Oklahoma State University, Stillwater, Oklahoma, United States of America; 3 Kearney Agricultural Research & Extension Center, Parlier, California, United States of America; University of Tours, FRANCE

## Abstract

House flies are of major concern as vectors of food-borne pathogens to food crops. House flies are common pests on cattle feedlots and dairies, where they develop in and feed on animal waste. By contacting animal waste, house flies can acquire human pathogenic bacteria such as *Escherichia coli* and *Salmonella* spp., in addition to other bacteria, viruses, or parasites that may infect humans and animals. The subsequent dispersal of house flies from animal facilities to nearby agricultural fields containing food crops may lead to pre-harvest food contamination with these pathogens. We hypothesized that odors from honeydew, the sugary excreta produced by sucking insects feeding on crops, or molds and fungi growing on honeydew, may attract house flies, thereby increasing the risk of food crop contamination. House fly attraction to honeydew-contaminated plant material was evaluated using a laboratory bioassay. House flies were attracted to the following plant-pest-honeydew combinations: citrus mealybug on squash fruit, pea aphid on faba bean plants, whitefly on navel orange and grapefruit leaves, and combined citrus mealybug and cottony cushion scale on mandarin orange leaves. House flies were not attracted to field-collected samples of lerp psyllids on eucalyptus plants or aphids on crepe myrtle leaves. Fungi associated with field-collected honeydews were isolated and identified for further study as possible emitters of volatiles attractive to house flies. Two fungal species, *Aureobasidium pullulans* and *Cladosporium cladosporioides*, were repeatedly isolated from field-collected honeydew samples. Both fungal species were grown in potato dextrose enrichment broth and house fly attraction to volatiles from these fungal cultures was evaluated. House flies were attracted to odors from *A*. *pullulans* cultures but not to those of *C*. *cladosporioides*. Identification of specific honeydew odors that are attractive to house flies could be valuable for the development of improved house fly baits for management of this pest species.

## Introduction

Food-borne pathogens including *Escherichia coli*, *Salmonella* spp., and *Campylobacter* spp. are persistent and widespread problems in food production and preparation [1–2], and the Centers for Disease Control (CDC) estimates that there are 48 million cases of food-borne illness in the United States each year [3]. Leafy vegetables were the second most common food commodity associated with Shiga toxin-producing *E*. *coli* outbreaks and are considered one of the top commodities for food-borne disease outbreaks [3]. Flies magnify the risk of food-borne disease by contacting animal waste, carrying pathogens, and subsequently dispersing throughout the surrounding area, transporting pathogens from places where they pose little risk to humans, such as at animal rearing facilities, to places where the risk is greatly amplified [4–5]. However, the extent to which flies contribute to the maintenance and spread of pathogens within and among livestock operations and the community is not well documented. Flies may play a key role in the distribution of human pathogens from domestic livestock to human food crops [6], but it is still unknown whether flies are attracted to human food crops or simply encounter them randomly during undirected movement through the environment.

Filth flies, including the house fly (*Musca domestica* L.), are closely associated with animal waste, from which they can acquire a variety of potentially pathogenic protozoa, viruses, and bacteria [7–9]. For example, in Japan, house flies were identified as the biotic factor most closely associated with the spread of pathogenic *E*. *coli* from livestock to children [10]. Furthermore, area-wide reductions in house fly abundance have been correlated with concurrent reductions in human illness due to enteric pathogens [11–15]. The dispersal of antibiotic resistant bacteria from animal and human waste to the environment is also of increasing concern [16–17].

In 2006, an outbreak of *Escherichia coli* O157:H7 associated with bagged spinach affected over 200 individuals in 26 US states [18]. Subsequent studies in the region where the contaminated spinach originated resulted in the capture of filth flies carrying this pathogen on leafy greens (spinach and lettuce) in field crops adjacent to a cattle pasture [6]. Filth flies may have contaminated pre-harvest leafy greens with human pathogens by deposition of fly feces and/or regurgitant onto the leaves [19]. However, filth flies are not typically associated with leafy green crops, and flies that typically develop in crop waste would not be expected to harbor human or animal pathogens such as *E*. *coli* O157:H7. Therefore, it is likely that *E*. *coli*-contaminated flies captured in leafy green crop fields had previous contact with *E*. *coli*-contaminated animal feces in a nearby cattle pasture [6].

Within a field of leafy greens, filth flies are more abundant where honeydew-producing insects are present [6]. The authors have observed house flies feeding on honeydew (S1 and S2 Figs), a carbohydrate-rich excretion produced by phloem-feeding insects [20], presumably to obtain the carbohydrates needed to sustain flight or other physiological functions [21]. House fly dispersal behavior is poorly understood, but odors from honeydew and/or fungi growing on honeydew-contaminated crop plants may attract flies. Alternatively, flies may simply accumulate on honeydew-contaminated crop plants while feeding on the available sugars, i.e., arrestment rather than attraction. Some insects orient toward honeydew odors as an indicator of food or oviposition sites [22–23]; however, honeydew sugars are not volatile or odorous. Thus, we hypothesized that house flies detect and orient toward honeydew by following odor plumes associated with honeydew, and that fungal digestion of honeydew sugars may produce such odors. Thus, our project objectives were:

1. To assess house fly attraction to odors from several insect honeydew and host-plant combinations, and
2. To assess house fly attraction to odors produced by cultures of two fungi repeatedly isolated from field-collected honeydew.

## Materials and Methods

### Insect colonies

House flies were collected by sweep net from a southern California dairy facility in 2010 with the owner’s permission and subsequently maintained as a colony in an insectary at constant conditions of 25 ± 2°C, 40% relative humidity (RH), and 12L:12D photoperiod. Immature flies were fed a standard fly rearing medium [24], and adult flies were fed a 50:50 mixture of powdered milk and sucrose, and given water *ad libitum*. Citrus mealybugs (*Planococcus citri* Risso) were from an established UC Riverside colony maintained on butternut squash fruit at 25 ± 2°C, 40% RH and 14L:10D photoperiod in an insectary room. Pesticide-free butternut squash grown in the Agricultural Operations fields at UC Riverside were harvested, thoroughly washed with a dilute solution of micro90 detergent, rinsed with water, dried for 1–2 days, and stored at 15.5°C until use (up to 1 year). Pea aphids (*Acyrthosiphon pisum* Harris) were established at UC Riverside in 2009 from a colony maintained at Oklahoma State University. Pea aphids were reared in a greenhouse on faba bean plants (*Vicia faba* L.) and maintained under ambient daylight conditions.

### Bioassays to assess house fly attraction

**Fig 1:**
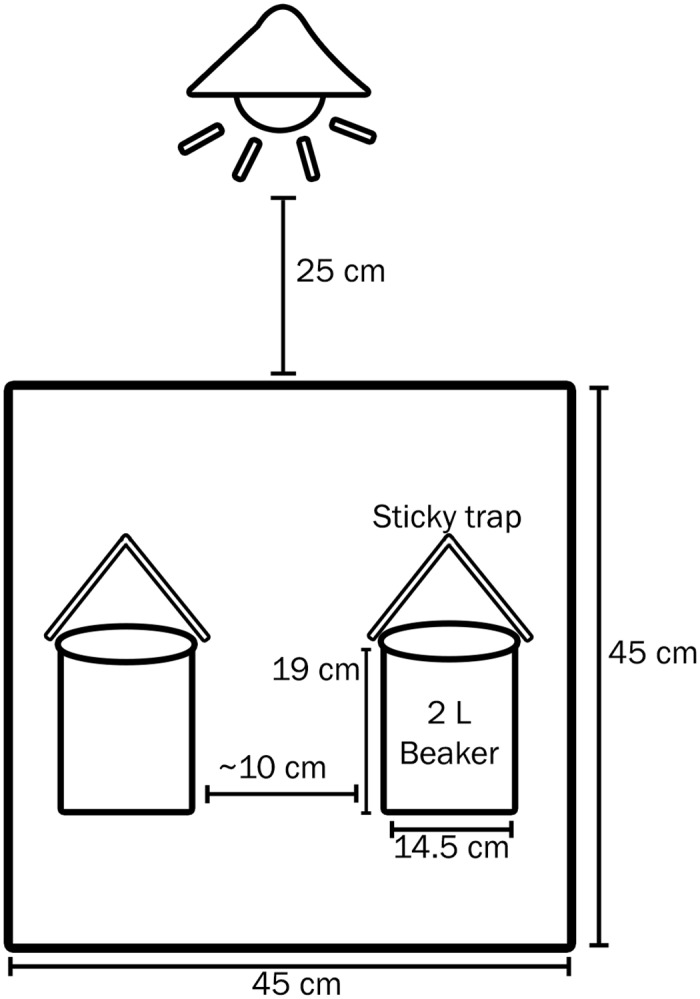
A diagram of a cage bioassay set up. Beakers containing either honeydew and plant material or a control were placed about 10 cm apart in a 45 × 45 × 45 cm cage. Sticky traps (sticky surface down) placed above each beaker captured flies near the beaker’s opening.

Bioassays were conducted in a room maintained at 25 ± 2°C, 40% RH, and 14L:10D photoperiod in the UC Riverside Insectary building. The ventilation system in the room provided a slight negative pressure with air pulled through a centrally positioned ceiling vent creating airflow throughout the room. Prior to initiating bioassays, the entire room was scrubbed with unscented soap and water, and floor drains were sealed to reduce outside odors. Eight screen-cages (45 × 45 × 45 cm, Product no. 1450D, BioQuip Products, Rancho Dominquez, CA, USA) were set up on wire shelving with a 57-watt incandescent bulb within an 20.3-cm aluminum reflector housing placed 25 cm above each cage to provide light that was evenly distributed across each cage. Each cage contained two 2-L glass beakers to hold test materials. Beakers were washed with micro90 detergent and rinsed thoroughly with deionized water to remove odors prior to use in bioassays. For each bioassay period, treatment and control materials were randomly assigned to a beaker position (left or right side of the cage) in cage #1, and the position of the treatment and control material was then alternated in cages #2–8 to minimize within-cage position effects. The beakers were placed approximately 10 cm apart from each other and covered with mesh netting held in place with a rubber band to prohibit fly access to material held within the beaker. White sticky cards (Pherocon 1C Liner, Trécé, Adair, OK, USA) were folded into a tent shape, with the sticky surface on the inside, and fixed above each beaker with a metal wire (Fig 1).

Flies (3–5 d-old) were removed by aspirator from colony cages, sorted on a chill table (Bioquip Products, Rancho Dominquez, CA, USA) into 8 groups of 50 female flies (400 flies total), and each group was placed into a release chamber (947 ml clear plastic food cup, First Street, Amerifoods Trading Co., Los Angeles, CA, USA) covered by a plastic lid with many small holes for air exchange and a 2.5 cm diameter hole blocked with a cork that could be easily removed allowing flies to exit. The release chamber contained a 30 mL plastic cup (First Street, Amerifoods Trading Co., Los Angeles, CA, USA) with water-soaked paper towel to provide moisture for the flies. Flies were starved in the bioassay room for 40–44 hr before use. The release chamber was used to slow the release of flies into the bioassay cage because early trials showed that a rapid release of flies into the assay cage resulted in substantial undirected flight and similar capture of flies on both treatment and control sticky cards within the first few minutes of the assay start time. An assay period thus began by placing a release chamber positioned equidistant from the treatment and control beakers into each of the 8 assay cages, followed by removal of the cork to allow starved flies to individually emerge from the release chambers during the 24 hr bioassay period, after which the number of flies captured on each sticky card was recorded.

### House fly attraction to honeydew from colony insects

Prior to use in an assay, clean butternut squash fruit were introduced into the citrus mealybug colony for at least 4 wk resulting in infestation of squash fruit by large numbers of nymph and adult mealybugs. This exposure period ensured sufficient production and deposition of mealybug honeydew onto the squash. Faba bean plants 30–45 cm tall were infested with ~ 50 pea aphids of different life stages and then held in the colony room for an additional exposure period of 2–3 wk to allow for sufficient production and deposition of aphid honeydew onto leaf surfaces. Uninfested faba bean plants were grown in a separate colony room under similar conditions and for the same 2–3 wk period. Following the aphid exposure period, both infested and uninfested faba bean plants were cut near the base of the stem for placement into beakers within the bioassay cages.

The following bioassay comparisons were made using colony insects: (1) Infested squash were compared to an empty beaker during 4 separate bioassay periods to evaluate variation in fly response by cage and by assay period; (2) Mealybug-infested squash were compared to uninfested squash during two bioassay periods, with both infested and uninfested squash incubated in the citrus mealybug colony room for the same 4 wk exposure period; (3) Uninfested squash were compared to empty beakers to evaluate fly attraction to the squash alone; (4) Uninfested squash punctured 30× with a needle to simulate insect feeding damage and then incubated in the mealybug colony room for 2 wks until signs of decay were obvious were compared to intact, uninfested squash to evaluate whether house flies were attracted to damaged and decaying fruit alone; 5) Pea aphid-infested faba bean plants were compared to uninfested faba bean plants (Table 1). Four cages were removed from comparison (1) and one cage was removed from comparison (4) due to flies accessing the beaker materials through gaps or tears in the mesh covering.

**Table 1:** House fly attraction to plant materials infested with honeydew-producing insects.

Treatment	Control	Treatment mean captures ± SEM	Control mean captures ± SEM	P-value	% Response to treatment	Reps
Citrus mealybug on squash	Nothing	13.68 ± 0.99	7.21 ± 0.48	p < 0.0001	65.5	28*
Citrus mealybug on squash	Uninfested squash	19.1 ± 1.34	12.6 ± 0.87	p = 0.0028	60.3	16
Uninfested squash	Nothing	8.75 ± 1.29	9.88 ± 1.38	p = 0.62	47.0	8
Needle-damaged squash	Uninfested squash	9.57 ± 2.77	8.43 ± 1.65	p = 0.79	53.2	7*
Pea aphid on faba bean plant	Faba bean plant, not infested	18.4 ± 1.34	10.3 ±1.26	p = 0.012	64.1	8

Fifty female flies were used per replicate, with 8 replicates per 24 hr bioassay period. Data were analyzed with ANOVA for the first comparison and by paired t-test for the remaining comparisons within each row. “% Response to treatment” is the total number of house flies captured on the treatment over the total flies captured on both treatment and control.

*Indicates that 1 or more replicates were discarded due to a cage failure allowing flies to access the test material within a beaker.

### Collection of honeydew and associated fungi from field sites

Plant stems and leaves with accumulated honeydew and without honeydew (control) were collected using latex or nitrile gloves to avoid contamination with human skin odors. All honeydew-contaminated and control samples were pruned from the plant with sterile shears, stored in oven bags (Terinex Ltd., Bedford, England) to maintain odors, and placed on ice for transport back to the laboratory for bioassays. Transport of plant material and honeydew was approved by the California Department of Food and Agriculture (permit #2776). Navel orange (*Citrus sinensis* Osbeck) and marsh grapefruit (Citrus paradisi *Macfadyen)* leaves infested with whiteflies (Family: Aleyrodidae) were collected from a single site in Riverside, CA. These two types of citrus plants were maintained as separate samples for fungal isolation but were combined together in equal amounts by mass for house fly attraction bioassays. Similarly, cottony cushion scale (*Icerya purchasi* Maskell) and citrus mealybugs on honey mandarin orange trees (Citrus reticulata *Blanco*) were collected from a single site at the UC Riverside Agricultural Operations citrus groves and maintained as separate samples for fungal isolation but were combined together for bioassay. Eucalyptus leaves (tentatively river red gum, *Eucalyptus camaldulensis* Dehnh) infested with lerp psyllids (*Glycaspis brimblecombei* Moore) were collected from a eucalyptus grove on the UC Riverside campus. Lerp psyllid honeydew was also collected on leaves from a red ironbark eucalyptus tree (*Eucalyptus sideroxylon* A. Cunn.) in Riverside, CA. Data from one cage was not included in analyses due to flies accessing the beaker materials. A second collection of foliage from the same red ironbark eucalyptus with lerp psyllid honeydew received ~20 mL of water in the collection bag and was incubated at 60% relative humidity and 25°C in the insectary room for one day before evaluation in the bioassay. Crepe myrtle (*Lagerstroemia* sp.) cuttings infested with an unidentified aphid species were collected from a residential yard with the owner’s permission in Riverside, CA. Because plant material and honeydew dries out quickly after collection, attraction of house flies to each of these field-collected honeydews was evaluated within 48 hr of collecting material from the field. If available, uninfested material from the same plant or a nearby plant of the same species was used in control beakers, otherwise control beakers contained nothing (Table 2).

**Table 2:** House fly attraction to field-collected plant materials contaminated with honeydew.

Treatment	Control	Treatment mean ± SEM	Control mean ± SEM	Paired t-test	% Response to treatment	Reps
Citrus mealybug and cottony cushion scale on honey mandarin	Honey mandarin cuttings, not infested	12.5 ± 0.63	8.5 ± 1.09	p = 0.009	59.5	8
Whitefly on marsh grapefruit and navel orange	Beaker only	11.6 ± 1.22	7.6 ±1.12	p = 0.029	60.4	8
Lerp psyllid on red ironbark eucalyptus	Red ironbark eucalyptus, not infested	14.9 ± 1.04	12.3 ±0.92	p = 0.13	54.8	8
Lerp psyllid on red ironbark eucalyptus incubated under high humidity 24 hr	Beaker only	7.9 ±0.77	6.0 ± 0.96	p = 0.15	56.8	8
Lerp psyllid on river red gum eucalyptus	River red gum eucalyptus, uninfested	7.7 ± 0.70	7.7 ±0.76	p = 1.0	50.0	7*
Aphids on crepe myrtle	Crepe myrtle, uninfested	20.3 ±2.68	16.9 ±1.64	p = 0.43	54.5	8

Fifty female flies were used per replicate, with a maximum of 8 replicates per 24 hr assay period. Data were analyzed by paired t-test for comparisons within each row. “% Response to treatment” is the total number of house flies captured on the treatment over the total flies captured on both treatment and control.

*One or more replicates were discarded due to a cage failure allowing flies to access the test material within a beaker.

For each honeydew-contaminated and uncontaminated (control) sample taken from the field, a small portion (13–33 g) was removed and washed with 15–30 mL deionized water (dH_2_O), where wash volume depended upon the volume of the field sample. Similar methods were used for host plant materials from citrus mealybug and pea aphid colonies, except that 8 week-old butternut squash infested with citrus mealybugs and clean butternut squash controls were gently washed with 30 mL of dH_2_O and the runoff was collected. Following their use in bioassays and for fungal collection, all plant material and honeydew residues were autoclaved before disposal.

The wash water from each sample was frozen and shipped overnight on ice to the Kearney Agricultural Research and Extension Center (KAREC), where 100 μl of leaf wash was diluted in sterile water as needed (1:10–1:1000) to obtain isolated colonies on a plate of growth medium. Thus, 100 μl of the dilution was applied to 4 Petri dishes containing acidified potato dextrose agar (APDA; 39 g potato dextrose agar acidified with 2.4 ml of a 25% v:v lactic acid per L medium) and incubated at 25°C for 5 d. The colony forming units (CFU) for each fungal species on the plates were counted, and the mean CFUs per plate were used to calculate the original concentration (CFU/100μl) in each wash sample by multiplying the colony counts by the dilution factor.

Fungi were identified to genus or species when possible by morphology and color. Polymerase chain reaction (PCR) and sequencing was used to verify species identification of *Aureobasidium* sp., *Cladosporium* sp., and *Rhodotorula* sp. Single-spore isolates were grown on 2% potato dextrose agar (PDA) for 7–10 d at 25±3°C for DNA extraction. Pure culture mycelia were scraped directly from the medium using a sterile scalpel and the total genomic DNA was extracted using a FastDNA Kit (BIO 101, Inc., Vista, CA, USA). The internal transcribed spacer (ITS) regions, including ITS1, ITS2, and the 5.8S rRNA gene of the ribosomal DNA (rDNA), were amplified using primers ITS1 and ITS4 [25]. The PCR of ITS regions were conducted according to previous studies [26–27]. An UltraClean PCR Clean-Up Kit (MO BIO Laboratories, Inc., Solana Beach, CA, USA) was used to purify the PCR products. The resulting amplicons were sequenced in both directions using the same primers used for the PCR reactions. Sequence reactions were run on an automated sequencer by the University of California-Davis, Division of Biological Sciences sequencing facility and the sequences were compared with the ex-type isolates of the fungal species for definitive identification. *Fusarium* sp. from the citrus mealybug colony was handled similarly as above except the DNA was extracted using an AllPrep DNA/RNA/miRNA Universal Kit (Cat. No. 80224, Qiagen Sciences, Germantown, MD, USA) and purified using Diffinity RapidTip (Cat. No. RT025-008, Diffinity Genomics, Inc., West Henrietta, NY, USA). The resulting amplicons were sequenced at the University of California, Riverside, Genomics Core sequencing facility using the ITS1 primer. The sequence was input into the NCBI’s BLAST sequence database and identified to 99% identity. Identified fungi were isolated and grown on individual APDA plates and stored at 4°C up to 6 mo or placed in 25% glycerol at -80°C for longer term storage.

In preparation for use in bioassays, isolated fungi from the honeydew-contaminated plant washes were plated onto APDA and shipped overnight to UC Riverside, then stored at 4°C until use. Autoclaved potato dextrose broth (PDB) media (250 mL) was inoculated with either *Aureobasidium pullulans* or *Cladosporium cladosporioides* and grown at 25°C under otherwise sterile conditions. *Cladosporium cladosporioides* was incubated for 2 wks while stirring constantly to achieve some degree of homogeneity throughout the media. *Aureobasidium pullulans* was incubated for 4 wks and allowed to grow as a layer on the surface of the media. About 30 mL aliquots of the *C*. *cladosporioides* culture or equal sized sections of the *A*. *pullulans* surface layer were placed into 8 glass petri dishes (9 cm diameter) that had been washed with micro90 detergent, rinsed with tap water, deionized water and acetone, then baked at 140°C to remove any residual odors. Petri dishes containing either the fungal cultures or sterile PDB (uninoculated and held under sterile conditions for the same incubation time) were placed into treatment or control glass beakers, respectively, for the bioassay as described above. House fly attraction to *C*. *cladosporioides* was evaluated during one bioassay period while attraction to *A*. *pullulans* was evaluated over two bioassay periods to confirm the significant attraction noted in the first bioassay period.

### Data Analysis

For bioassay comparison #1 (mealybug-infested squash vs. nothing) the number of flies captured on each sticky trap (treatment and control) during each bioassay period was log transformed to normalize the data and then analyzed for differences among assay cage, assay period, and treatment (and for interactions among these factors) using multi-factorial ANOVA with treatment means separated using Fisher’s least significant difference (LSD) test. All remaining bioassay comparisons were analyzed by calculating the difference between the number of flies captured on the sticky cards above the treatment and the control in each cage (diff = treatment - control) and using a one sample t-test to compare the observed difference values to an expected value of zero, if the treatment and control were not different in their attraction to flies. The Shapiro-Wilk test was used to verify normality of all data sets prior to analysis. All analyses were performed using SAS statistical software v9.4 (Cary, NC, USA).

## Results

### House fly attraction to laboratory-reared honeydew-producing insects

House flies were significantly more attracted to mealybug-infested squash relative to the empty beaker control (F = 72.87; df = 1,55; p < 0.0001) (Table 1). There were significant differences in the total number of flies captured among assay periods (F = 12.6; df = 3,55; p = 0.0001) indicating variable participation by each cohort of flies used, but no differences among assay cages (F = 1.43; df = 7,55; p = 0.26) and no interactions between treatment and assay period (F = 2.53; df = 3,55; p = 0.092) or treatment and assay cage (F = 1.42; df = 7,55; p = 0.26). Thus, we deemed the bioassay suitable for evaluation of house fly responses to odors.

House flies were significantly attracted to mealybug-infested squash relative to uninfested squash (t = 3.56; df = 1,15; p = 0.0028), but not to uninfested squash relative to an empty beaker (t = 0.51; df = 1,7; p = 0.62) or to needle-damaged, decaying squash relative to uninfested/undamaged squash (t = 0.28; df = 1,6; p = 0.79). Flies were also attracted to aphid-infested faba bean plants relative to uninfested faba bean plants (t = 3.39; df = 1,7; p = 0.012).

### House fly attraction to field-collected host plants infested with sucking insects

House flies were attracted to combined samples of whitefly-infested orange and grapefruit leaves (t = 2.73; df = 1,7; p = 0.029) and to citrus mealybug and cottony cushion scale-infested mandarin orange leaves (t = 3.58; df = 1,7; p = 0.009) (Table 2). In contrast, flies were not attracted to lerp psyllid-infested foliage of red ironbark eucalyptus (t = 1.7; df = 1,7; p = 0.13). Wetting the lerps on red ironbark eucalyptus foliage followed by incubation for 24 hr under humid conditions did not increase fly attraction (t = 1.6; df = 1,7; p = 0.15). Flies were also not attracted to aphid-infested crepe myrtle (t = 0.85; df = 1,7; p = 0.43) or to lerp psyllid-infested red river gum eucalyptus leaves (t = 0.00; df = 1,6; p = 1.0).

### House fly attraction to volatiles of fungi isolated from honeydew-contaminated plants

*Aureobasidium pullulans* and *C*. *cladosporioides* were identified in higher concentrations from most of the field collected honeydew samples relative to the corresponding uninfested (control) plant material. Neither fungus was detected from the laboratory cultures of citrus mealybug on squash, where *Fusarium solani* was predominant (Table 3). Other fungi isolated from wash water samples of field-collected, honeydew-contaminated plant materials included *Rhodotorula* sp., *Penicillium* sp., *Alternaria alternata*, *Fusarium* sp., *Aspergillus niger*, and *Rhizopus stolonifer*. However the isolation of these fungi was inconsistent across honeydew samples or their concentrations in the honeydew samples were similar to the concentrations in wash samples from uninfested plant material. House flies were significantly attracted to odors from *A*. *pullulans* cultured in PDB (t = 3.62; df = 1,15; p = 0.0025) but not to odors from *C*. *cladosporioides* in PDB (t = 0.64; df = 1,7; p = 0.54) relative to sterile broth (Table 4).

**Table 3:** Fungi collected from honeydew-contaminated plant material.

		*Aureobasidium pullulans*		*Cladosporium cladosporioides*		*Rhodotorula spp*.		*Penicillium spp*.		*Alternaria alternata*		*Fusarium spp*.		*Aspergillus niger*	
Host plant	Insect	Tmt	Ct	Tmt	Ct	Tmt	Ct	Tmt	Ct	Tmt	Ct	Tmt	Ct	Tmt	Ct
Butternut squash fruit	Citrus mealybug colony	0	0	0	0	0	0	0	0	0	0	55000	0	0	0
Faba bean leaves	Pea aphid colony	26	0	109	15	11	0	5	0.25	0	0	7.5	0	6	1
Navel Orange Leaves	Whitefly	6490	450	1030	12	0	0	70	6	30	18	30	8	0	0
Grapefruit Leaves	Whitefly	>30000	680	>10000	128	0*	0	0*	0	0*	20	0*	0	0*	0
Mandarin leaves	Citrus Mealybug	4400	0	556	44	0	0	0	0	0	0	0	0	0	0
Mandarin leaves	Cottony cushion scale	9872	0	1294	44	0	0	0	0	0	0	0	0	0	0
Eucalyptus, red ironbark leaves	Lerp psyllid	347	58	38	0	0	0	256	0	13	15	0	0	0	0
Eucalyptus, river red leaves	Lerp psyllid	3625	1195	6	5	0	0	0	0	0	1	0	0	0	0
Crepe myrtle leaves	Aphid	>225000	207	2	1	0	0	0	0	1	0	0	1	0	0

Numbers are presented as colony forming units/100 μl or CFU/100ul. Rows above the double line are the honeydew-contaminated plant materials that were attractive to house flies. Rows below the double line were materials that were not attractive to flies. “Tmt” is treatment leaves or fruit containing insects and honeydew. “Ct” is control leaves or fruit with no visible insects or honeydew.

* Indicates there was overgrowth of the *Aureobasidium* and *Cladosporium* so that other specimens could not be identified.

**Table 4:** House fly attraction to odors from fungi isolated from honeydew and cultured on potato dextrose broth (PDB).

Treatment	Control	Treatment mean captures ± SEM	Ctrl mean captures± SEM	Paired t-test	% Response to treatment	Reps
*Aureobasidium pullulans*	PDB only	9.75 ± 0.78	6.06 ± 0.61	p = 0.0025	61.7	16
*Cladosporium cladosporioides*	PDB only	13.25 ± 1.76	11.63 ± 1.03	p = 0.54	53.3	8

Fifty female flies were used per replicate, with 8 replicates tested per 24 hr-assay period. Data were analyzed by paired t-test for comparisons in each row. “% Response to treatment” is the total number of house flies captured on the treatment over the total flies captured on both treatment and control.

## Discussion

House flies were attracted to odors associated with honeydew produced by a range of honeydew-producing insects, including citrus mealybug, pea aphid, whitefly, and cottony-cushion scale. To our knowledge, this is the first study to demonstrate house fly attraction to odors associated with honeydew, although house fly feeding on honeydew has previously been reported [20]. The honeydew-contaminated plant materials that were attractive to house flies were generally wet and sticky, with microbial growth on the honeydew deposits. In contrast, the non-attractive lerp psyllid-infested eucalyptus leaves were dry and showed no visible evidence of fungal or microbial growth on the leaves or on the crystallized honeydew forming the “lerps”. Whereas the authors have observed house flies feeding on lerps in the field (S2 Fig), the flies may have encountered the lerps during undirected movement rather than being attracted to them from a distance. Strong odors from the eucalyptus leaves may also repel flies or disrupt their detection of honeydew odors. Incubating lerps on eucalyptus leaves under high humidity conditions for 24 hr prior to conducting bioassays did not increase fly attraction, possibly due to insufficient time for fungi or other microorganisms to colonize the recently wetted lerp honeydew. We made no attempt to test fly attraction to lerp honeydew wetted for a longer period of time given the focus of this study on attraction to honeydews recently obtained from a field site. Although the aphids infesting crepe myrtle leaves released wet, sticky honeydew and leaves had some visible mold growth, the amount of honeydew and level of aphid infestation was relatively low, which may explain the lack of house fly attraction to this test substrate.

It is difficult to separate attraction to honeydew from attraction to honeydew-producing insects when evaluating house fly responses to field collected honeydews, because these samples often included some honeydew-producing insects or insect detritus. However, attraction solely to host plant volatiles was not noted in this study. In the absence of any honeydew, house flies were not attracted to volatiles of either intact squash or needle-damaged, decaying squash as a surrogate for an insect-damaged host plant. Furthermore, most bioassays used uninfested plant material as the control suggesting that flies were attracted to odors from honeydew-producing insects, honeydew, fungi or other honeydew colonizers, or to some combination of these rather than to plant volatiles alone. Nevertheless, enhancement of house flies attraction to honeydew odors as a result of the presence of host plant volatiles cannot be excluded.

Honeydew is rich in simple and complex carbohydrates [28], and is known to be a food source for dipteran species [29–34]. However, attraction of insect species in general to odors associated with honeydew has received relatively little study. Aphid honeydew is known to attract some aphid predators including the Asian lady beetle, *Harmonia axyridis* (Pallas), [23] and the aphidophagous gall midge, *Aphidoletes aphidimyza* (Rondani) [35]. Honeydew also stimulates oviposition by the aphidophagous hoverfly, *Episyrphus balteatus* (De Geer) [36]. Thus, for aphid parasitoids and predators at least, honeydew or odors associated with honeydew appear to serve as general kairomones for predation or oviposition. On the other hand, honeydew was not attractive to wild *Aedes albopictus* (Skuse) mosquitoes when tested under field conditions [37] despite observations of mosquitoes feeding on honeydew in the field [32].

Two fungi (*A*. *pullulans* and *C*. *cladosporioides*) were repeatedly isolated from field-collected plant materials contaminated with honeydew that were attractive to house flies in laboratory bioassays. Volatile odors from one of these fungi (*A*. *pullulans*) grown on PDB media were attractive to house flies in the absence of any honeydew or insect odors, suggesting that house fly attraction to honeydew may be due, at least in part, to odors produced by this fungus. These odors are likely byproducts from the metabolic breakdown of the honeydew by the fungi [38]. In a related example, odors produced by bacteria isolated from honeydew have been shown to attract syrphid flies [39].

*Aureobasidium pullulans* is a yeast-like fungus which is commonly found in soil and on plant surfaces. It also constitutes the dark sooty mold that colonizes leaves and is aptly named “black yeast” [40]. Odors from *A*. *pullulans* have been previously shown to attract *Vespula* spp. wasps [41] and increase the number (particularly Diptera) and diversity of insects captured in an agricultural field [42]. Because of the ubiquity of this fungus as a colonizer of insect honeydews, odors resulting from metabolism of honeydew by *A*. *pullulans* might signal the availability of a carbohydrate-rich food source. Fermentation odors produced by *A*. *pullulans* may therefore be general insect attractants, as suggested by Davis and Landolt [42].

The lack of house fly attraction to odors from the fungus *C*. *cladosporioides* grown on PDB does not prove that house flies would not be attracted to volatiles produced by this fungus when growing on honeydew under field conditions. In particular, odors produced as a byproduct of metabolism may vary depending upon the media on which microbial species are grown [43]. Interestingly, the concentration of *C*. *cladosporioides* was low in the non-attractive honeydew from aphids on crepe myrtle leaves and from lerp psyllids on eucalyptus leaves, whereas the concentration of *C*. *cladosporioides* was very high in the attractive honeydew from whiteflies on marsh grapefruit and on navel orange, cottony cushion scale and citrus mealybugs on honey mandarin, and pea aphids on faba bean. This suggests that odors produced by *C*. *cladosporioides* may act synergistically with *A*. *pullulans* odors, or that *C*. *cladosporioides* needs specific substrates or different growing conditions in order to produce odors attractive to house flies.

In contrast, butternut squash infested with citrus mealybugs that attracted flies in laboratory bioassays did not yield either *A*. *pullulans* nor *C*. *cladosporioides*, but was heavily colonized by *F*. *solani*. It remains to be determined whether the attraction seen was due to *F*. *solani* producing a volatile profile similar to *A*. *pullulans*, or whether the two fungal species have markedly different, but still attractive, odor profiles.

Overall, the results from this study support our hypothesis that honeydew production by sucking insects infesting food crops may contribute to attraction of house flies to those crops, particularly when they are grown in proximity to animal rearing facilities or other sites that produce large numbers of house flies. Thus, managing honeydew-producing insects on food crops may reduce house fly visitation, and consequently the risk of crop contamination with food-borne pathogens.

Growing concerns about insecticide resistance and the environmental impact of chemical insecticides are driving the search for alternative pest control methods, including “attract and kill” strategies using attractive odors to draw pest insects into traps [44]. A few studies have identified odors that are attractive to house flies, most of which are odors associated with animal feces or are products of fermentation or decay [45–50]. Some of these odors are unpleasant to humans and may target female flies seeking a protein source or oviposition site rather than a sugar-based food source. Although levels of fly attraction to plant materials contaminated with insects and honeydew versus controls in this study was modest, with 60–65% of flies captured at honeydew, the isolation and identification of specific honeydew odors attractive to house flies could provide useful alternate attractants for comparison with currently known attractants.

## Supporting Information

S1 Fig House flies feeding on soft scale honeydew. Honeydew was produced by soft scales infesting jacaranda trees in Chino, CA. This location is less than 1 mile from an agricultural animal facility. Image was taken by ACG.
(JPG)

S2 Fig House flies feeding on “lerp” honeydew. Honeydew was produced by lerp psyllids infesting unidentified eucalyptus trees in Bakersfield, CA. These trees were in the proximity of an animal agricultural facility. Image was taken by ACG.
(JPG)
