# Supplementary figures and images for: House Fly (Musca domestica L.) Attraction to Insect Honeydew
Source: PLoS One. 2015 May 13;10(5):e0124746. doi: 10.1371/journal.pone.0124746 (PMC4430494; doi:10.1371/journal.pone.0124746)

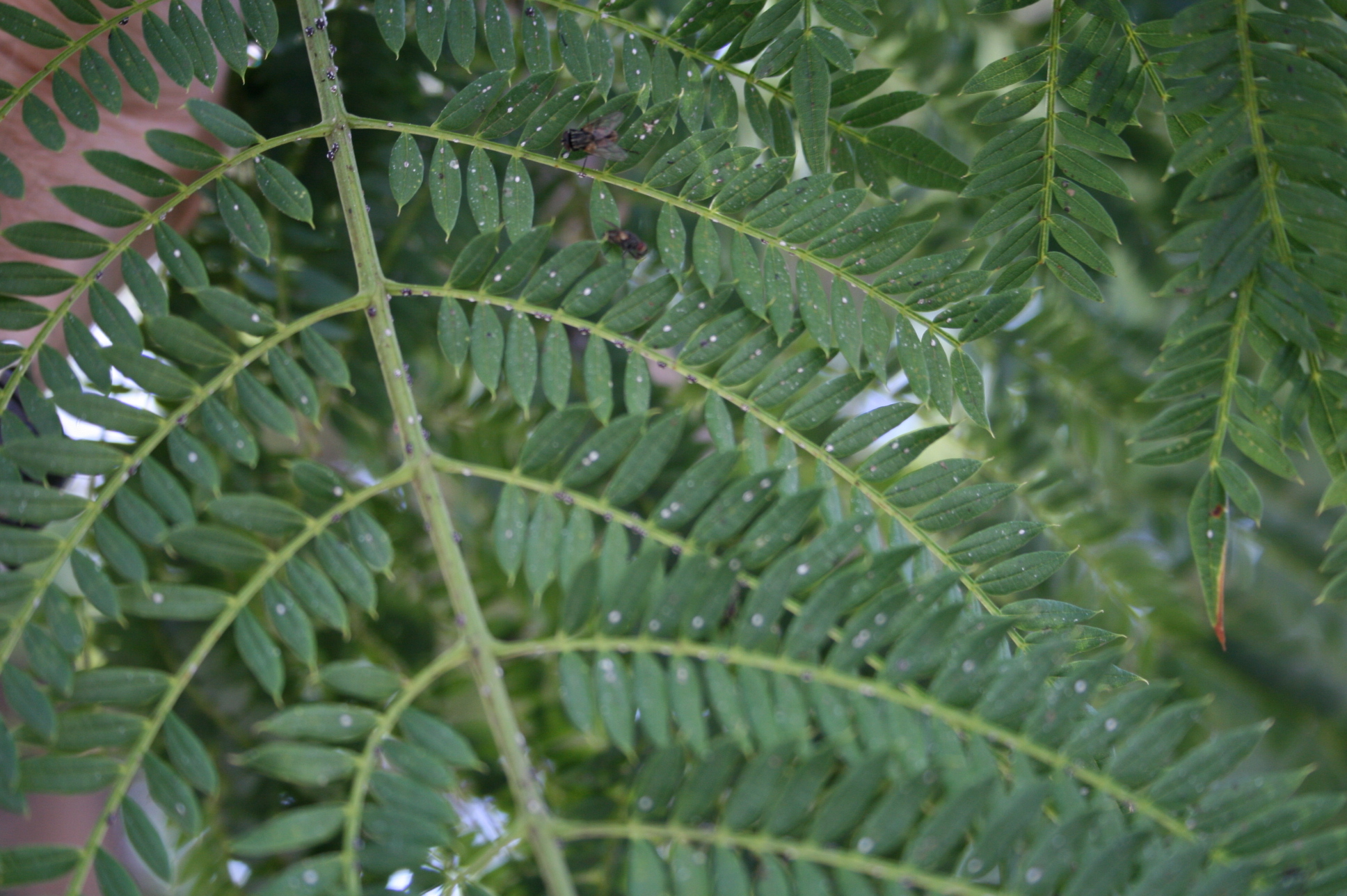

Supplement: S1 Fig — Honeydew was produced by soft scales infesting jacaranda trees in Chino, CA. This location is less than 1 mile from an agricultural animal facility. Image was taken by ACG. (JPG) [file pone.0124746.s001.jpg]

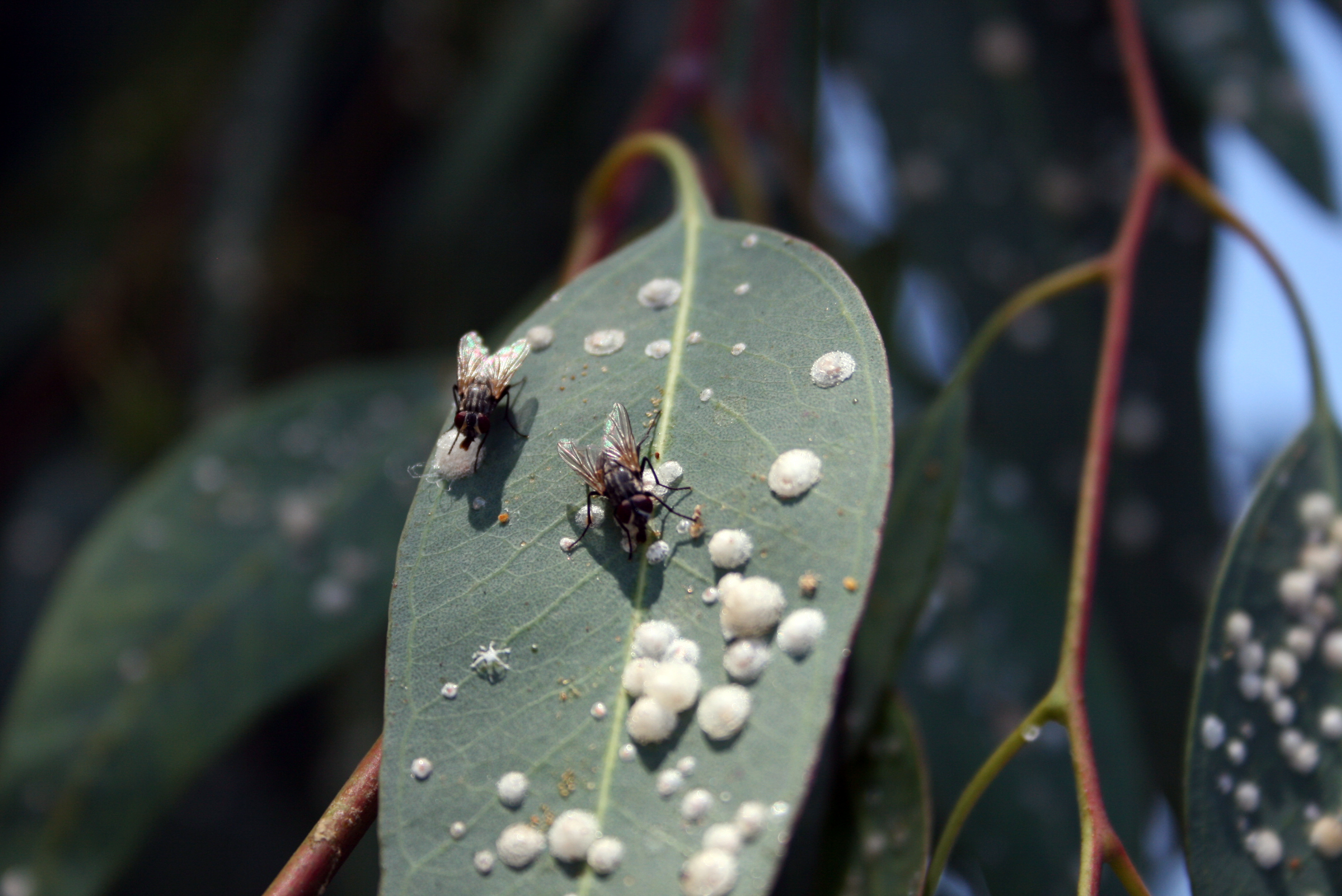

Supplement: S2 Fig — Honeydew was produced by lerp psyllids infesting unidentified eucalyptus trees in Bakersfield, CA. These trees were in the proximity of an animal agricultural facility. Image was taken by ACG. (JPG) [file pone.0124746.s002.jpg]
